# 2-(Tritylsulfan­yl)ethyl 3-iodo­benzoate

**DOI:** 10.1107/S1600536811036427

**Published:** 2011-09-14

**Authors:** Xin Zhu, Seik Weng Ng

**Affiliations:** a Henan University of Traditional Chinese Medicine, Zhengzhou 450008, People’s Republic of China; b Department of Chemistry, University of Malaya, 50603 Kuala Lumpur, Malaysia, and, Chemistry Department, King Abdulaziz University, PO Box 80203 Jeddah, Saudi Arabia

## Abstract

The triphenyl­methyl group in the title compound, C_28_H_23_IO_2_S, has the methine carbon slightly flattened out [ΣC_phen­yl_—C—C_phen­yl_ = 332.8 (6) °]. The –C–O–C–C–S– chain connecting the triphenyl­methyl group and the aromatic ring adopts an extended zigzag conformation, these five atoms being approximately co-planar (r.m.s. deviation 0.260 Å).

## Related literature

For the 2-iodo­benzoate analog, see: Zhu *et al.* (2011 ▶).
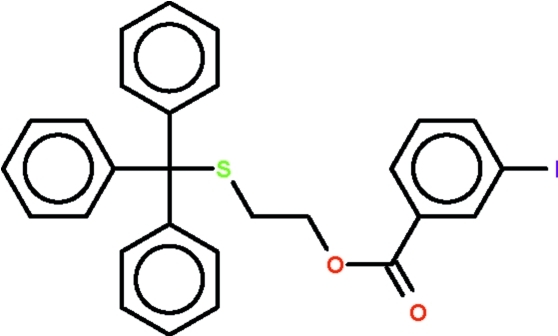


## Experimental

### 

#### Crystal data

C_28_H_23_IO_2_S
*M* *_r_* = 550.42
Triclinic, 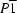

*a* = 8.1634 (8) Å
*b* = 8.8413 (9) Å
*c* = 18.9968 (18) Å
α = 89.493 (1)°
β = 79.270 (2)°
γ = 65.663 (1)°
*V* = 1223.9 (2) Å^3^
*Z* = 2
Mo *K*α radiation
μ = 1.42 mm^−1^
*T* = 293 K
0.40 × 0.35 × 0.20 mm

#### Data collection

Bruker SMART APEX diffractometer
Absorption correction: multi-scan (*SADABS*, Sheldrick, 1996 ▶) *T*_min_ = 0.601, *T*_max_ = 0.765
5948 measured reflections
4254 independent reflections
3375 reflections with *I* > 2σ(*I*)
*R*_int_ = 0.018

#### Refinement

*R*[*F* ^2^ > 2σ(*F* ^2^)] = 0.045
*wR*(*F* ^2^) = 0.126
*S* = 1.02
4254 reflections
290 parameters
H-atom parameters constrained
Δρ_max_ = 0.89 e Å^−3^
Δρ_min_ = −1.33 e Å^−3^

### 

Data collection: *APEX2* (Bruker, 2007 ▶); cell refinement: *SAINT* (Bruker, 2007 ▶); data reduction: *SAINT*; program(s) used to solve structure: *SHELXS97* (Sheldrick, 2008 ▶); program(s) used to refine structure: *SHELXL97* (Sheldrick, 2008 ▶); molecular graphics: *X-SEED* (Barbour, 2001 ▶); software used to prepare material for publication: *publCIF* (Westrip, 2010 ▶).

## Supplementary Material

Crystal structure: contains datablock(s) global, I. DOI: 10.1107/S1600536811036427/zs2142sup1.cif

Structure factors: contains datablock(s) I. DOI: 10.1107/S1600536811036427/zs2142Isup2.hkl

Supplementary material file. DOI: 10.1107/S1600536811036427/zs2142Isup3.cml

Additional supplementary materials:  crystallographic information; 3D view; checkCIF report

## Figures and Tables

**Fig. 1.**
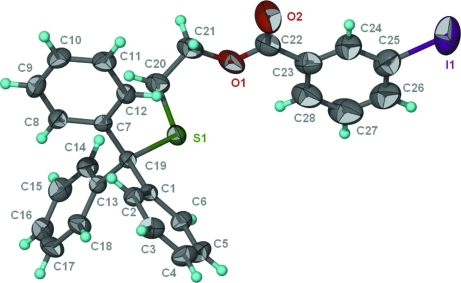
Thermal ellipsoid plot (Barbour, 2001) of C28H23IO2S at the 50% probability level. Hydrogen atoms are drawn as spheres of arbitrary radius.

## supplementary crystallographic information

### Comment

Triphenylmethyl is an important *S*-protecting group that prevents a thiol group from reacting with sensitive functional groups. The compound C_28_H_23_IO_2_S (Scheme I) was synthesized for the purpose of examining copper(I) chloride-catalyzed cleavage reactions, the present study following a previous structural determination of the 2-iodobenzoate analog (Zhu *et al.*, 2011). The methine carbon is slightly flattened out (ΣC_phenyl_–*C*–C_phenyl_ 332.8 (6) °) owing to decreased crowding by the S atom. The –C–O–C–C–S– chain connecting the triphenylmethyl group and the aromatic ring adopts an extended zigzag conformation, these five atoms lying on an approximate plane (r.m.s. deviation 0.260 Å) (Fig. 1).

### Experimental

A solution of 3-iodobenzoic acid (0.74 g, 3 mmol), dicyclohexylcarbodiimide (1.03 g, 5 mmol) and 4-dimethylaminopyridine (0.61 g, 8 mmol) in THF (20 ml) was stirred for an hour. 2-(tritylthio)ethanol (0.96 g, 3 mmol) was added. The reaction was stirred for 36 h. The compound was purified by column chromatography with petroleum ether–acetone (2:1) as the eluent and was isolated upon evaporation of the solvent as yellow crystals (2.30 g, 80% yield).

### Refinement

Carbon-bound H-atoms were placed in calculated positions (C—H = 0.93 to 0.97 Å) and were included in the refinement in the riding model approximation, with *U*_iso_(H) set to 1.2*U*_eq_(C). The final difference Fourier map had a peak in the vicinity of I1.

### Crystal data

**Table:**

C_28_H_23_IO_2_S	*Z* = 2
*M**_r_* = 550.42	*F*(000) = 552
Triclinic, *P*1	*D*_x_ = 1.494 Mg m^−^^3^
Hall symbol: -P 1	Mo *K*α radiation, λ = 0.71073 Å
*a* = 8.1634 (8) Å	Cell parameters from 2381 reflections
*b* = 8.8413 (9) Å	θ = 2.9–25.0°
*c* = 18.9968 (18) Å	µ = 1.42 mm^−^^1^
α = 89.493 (1)°	*T* = 293 K
β = 79.270 (2)°	Prism, yellow
γ = 65.663 (1)°	0.40 × 0.35 × 0.20 mm
*V* = 1223.9 (2) Å^3^	

### Data collection

**Table:**

Bruker SMART APEX diffractometer	4254 independent reflections
Radiation source: fine-focus sealed tube	3375 reflections with *I* > 2σ(*I*)
graphite	*R*_int_ = 0.018
ω scans	θ_max_ = 25.0°, θ_min_ = 2.7°
Absorption correction: multi-scan (*SADABS*, Sheldrick, 1996)	*h* = −9→9
*T*_min_ = 0.601, *T*_max_ = 0.765	*k* = −10→10
5948 measured reflections	*l* = −22→19

### Refinement

**Table:**

Refinement on *F*^2^	Secondary atom site location: difference Fourier map
Least-squares matrix: full	Hydrogen site location: inferred from neighbouring sites
*R*[*F*^2^ > 2σ(*F*^2^)] = 0.045	H-atom parameters constrained
*wR*(*F*^2^) = 0.126	*w* = 1/[σ^2^(*F*_o_^2^) + (0.0509*P*)^2^ + 1.4533*P*] where *P* = (*F*_o_^2^ + 2*F*_c_^2^)/3
*S* = 1.02	(Δ/σ)_max_ = 0.001
4254 reflections	Δρ_max_ = 0.89 e Å^−^^3^
290 parameters	Δρ_min_ = −1.33 e Å^−^^3^
0 restraints	Extinction correction: *SHELXL*, Fc^*^=kFc[1+0.001xFc^2^λ^3^/sin(2θ)]^-1/4^
Primary atom site location: structure-invariant direct methods	Extinction coefficient: 0.0354 (19)

### Fractional atomic coordinates and isotropic or equivalent isotropic displacement parameters (Å^2^)

**Table:**

	*x*	*y*	*z*	*U*_iso_*/*U*_eq_	
I1	0.70737 (7)	0.61227 (5)	0.60081 (2)	0.1219 (3)	
S1	0.73375 (12)	−0.09689 (11)	0.27224 (4)	0.0482 (2)	
O1	0.9395 (4)	−0.0436 (4)	0.37588 (14)	0.0733 (8)	
O2	1.0491 (6)	0.1484 (5)	0.3642 (2)	0.1083 (13)	
C1	0.5442 (4)	−0.0134 (3)	0.16643 (17)	0.0376 (7)	
C2	0.5437 (4)	0.0440 (4)	0.09843 (18)	0.0446 (7)	
H2	0.6475	−0.0058	0.0623	0.054*	
C3	0.3903 (5)	0.1748 (5)	0.0836 (2)	0.0569 (9)	
H3	0.3920	0.2127	0.0378	0.068*	
C4	0.2358 (5)	0.2489 (5)	0.1365 (2)	0.0626 (10)	
H4	0.1326	0.3365	0.1265	0.075*	
C5	0.2343 (5)	0.1929 (4)	0.2040 (2)	0.0591 (10)	
H5	0.1303	0.2440	0.2400	0.071*	
C6	0.3854 (4)	0.0617 (4)	0.21896 (19)	0.0482 (8)	
H6	0.3815	0.0227	0.2646	0.058*	
C7	0.8838 (4)	−0.1952 (4)	0.12723 (16)	0.0362 (6)	
C8	0.9698 (4)	−0.3353 (4)	0.08007 (18)	0.0455 (7)	
H8	0.9232	−0.4155	0.0825	0.055*	
C9	1.1256 (5)	−0.3583 (5)	0.0288 (2)	0.0576 (9)	
H9	1.1830	−0.4543	−0.0022	0.069*	
C10	1.1947 (5)	−0.2414 (5)	0.0235 (2)	0.0565 (9)	
H10	1.2975	−0.2564	−0.0115	0.068*	
C11	1.1113 (5)	−0.1011 (5)	0.0702 (2)	0.0549 (9)	
H11	1.1589	−0.0216	0.0673	0.066*	
C12	0.9576 (4)	−0.0776 (4)	0.12155 (18)	0.0462 (8)	
H12	0.9024	0.0180	0.1528	0.055*	
C13	0.6642 (4)	−0.3178 (4)	0.18842 (17)	0.0385 (7)	
C14	0.7540 (5)	−0.4510 (4)	0.2265 (2)	0.0510 (8)	
H14	0.8427	−0.4480	0.2502	0.061*	
C15	0.7132 (5)	−0.5887 (4)	0.2296 (2)	0.0601 (10)	
H15	0.7749	−0.6769	0.2555	0.072*	
C16	0.5843 (5)	−0.5972 (4)	0.1953 (2)	0.0625 (10)	
H16	0.5566	−0.6895	0.1982	0.075*	
C17	0.4959 (6)	−0.4673 (5)	0.1566 (3)	0.0659 (11)	
H17	0.4090	−0.4722	0.1323	0.079*	
C18	0.5353 (5)	−0.3291 (4)	0.1533 (2)	0.0557 (9)	
H18	0.4738	−0.2420	0.1269	0.067*	
C19	0.7098 (4)	−0.1647 (4)	0.18310 (16)	0.0364 (6)	
C20	0.9669 (5)	−0.2308 (5)	0.2817 (2)	0.0594 (9)	
H20A	0.9645	−0.3123	0.3160	0.071*	
H20B	1.0386	−0.2898	0.2359	0.071*	
C21	1.0527 (6)	−0.1264 (6)	0.3073 (2)	0.0700 (11)	
H21A	1.1769	−0.1958	0.3127	0.084*	
H21B	1.0567	−0.0452	0.2731	0.084*	
C22	0.9472 (6)	0.0942 (6)	0.3973 (2)	0.0688 (11)	
C23	0.8168 (6)	0.1760 (6)	0.4656 (2)	0.0694 (11)	
C24	0.8162 (6)	0.3195 (6)	0.4935 (2)	0.0754 (12)	
H24	0.8964	0.3620	0.4696	0.090*	
C25	0.6974 (7)	0.4010 (7)	0.5568 (2)	0.0841 (14)	
C26	0.5784 (8)	0.3367 (10)	0.5919 (3)	0.1035 (19)	
H26	0.4970	0.3913	0.6342	0.124*	
C27	0.5801 (8)	0.1935 (11)	0.5645 (3)	0.114 (2)	
H27	0.5005	0.1506	0.5886	0.137*	
C28	0.6994 (7)	0.1116 (8)	0.5013 (2)	0.0902 (16)	
H28	0.7003	0.0140	0.4829	0.108*	

### Atomic displacement parameters (Å^2^)

**Table:**

	*U*^11^	*U*^22^	*U*^33^	*U*^12^	*U*^13^	*U*^23^
I1	0.1472 (5)	0.1016 (4)	0.0882 (3)	−0.0278 (3)	−0.0128 (3)	−0.0138 (2)
S1	0.0494 (5)	0.0535 (5)	0.0438 (5)	−0.0241 (4)	−0.0075 (4)	−0.0034 (4)
O1	0.085 (2)	0.115 (2)	0.0464 (15)	−0.0680 (19)	−0.0136 (14)	0.0030 (15)
O2	0.131 (3)	0.129 (3)	0.082 (2)	−0.093 (3)	0.032 (2)	−0.024 (2)
C1	0.0354 (15)	0.0313 (15)	0.0498 (18)	−0.0184 (13)	−0.0062 (13)	0.0012 (13)
C2	0.0424 (17)	0.0432 (17)	0.0494 (19)	−0.0198 (15)	−0.0074 (14)	0.0049 (14)
C3	0.054 (2)	0.056 (2)	0.065 (2)	−0.0259 (18)	−0.0183 (18)	0.0207 (18)
C4	0.045 (2)	0.045 (2)	0.092 (3)	−0.0110 (16)	−0.018 (2)	0.0169 (19)
C5	0.0410 (19)	0.047 (2)	0.077 (3)	−0.0113 (16)	0.0002 (17)	0.0031 (18)
C6	0.0423 (18)	0.0459 (18)	0.053 (2)	−0.0179 (15)	−0.0026 (15)	0.0062 (15)
C7	0.0326 (15)	0.0367 (15)	0.0403 (16)	−0.0152 (12)	−0.0078 (12)	0.0046 (12)
C8	0.0420 (17)	0.0408 (17)	0.0540 (19)	−0.0196 (14)	−0.0045 (15)	0.0006 (14)
C9	0.050 (2)	0.053 (2)	0.059 (2)	−0.0162 (17)	0.0039 (17)	−0.0092 (17)
C10	0.0441 (19)	0.065 (2)	0.058 (2)	−0.0262 (17)	0.0034 (16)	0.0054 (18)
C11	0.051 (2)	0.055 (2)	0.070 (2)	−0.0343 (17)	−0.0078 (17)	0.0108 (18)
C12	0.0462 (18)	0.0416 (17)	0.055 (2)	−0.0243 (15)	−0.0048 (15)	−0.0004 (14)
C13	0.0369 (15)	0.0309 (15)	0.0475 (17)	−0.0172 (13)	−0.0005 (13)	0.0001 (12)
C14	0.0467 (19)	0.0408 (18)	0.067 (2)	−0.0198 (15)	−0.0118 (16)	0.0074 (16)
C15	0.061 (2)	0.0365 (18)	0.079 (3)	−0.0205 (17)	−0.006 (2)	0.0131 (17)
C16	0.061 (2)	0.0404 (19)	0.089 (3)	−0.0312 (18)	0.003 (2)	−0.0027 (18)
C17	0.064 (2)	0.055 (2)	0.095 (3)	−0.038 (2)	−0.023 (2)	0.002 (2)
C18	0.058 (2)	0.0453 (19)	0.076 (3)	−0.0291 (17)	−0.0241 (19)	0.0108 (17)
C19	0.0361 (15)	0.0337 (15)	0.0412 (16)	−0.0173 (13)	−0.0058 (12)	0.0012 (12)
C20	0.055 (2)	0.071 (2)	0.058 (2)	−0.0274 (19)	−0.0214 (18)	0.0077 (18)
C21	0.064 (2)	0.106 (3)	0.054 (2)	−0.048 (2)	−0.0177 (19)	0.004 (2)
C22	0.069 (3)	0.097 (3)	0.052 (2)	−0.048 (2)	−0.010 (2)	0.004 (2)
C23	0.064 (2)	0.105 (3)	0.047 (2)	−0.041 (2)	−0.0158 (19)	0.011 (2)
C24	0.074 (3)	0.097 (3)	0.054 (2)	−0.037 (3)	−0.007 (2)	0.007 (2)
C25	0.078 (3)	0.107 (4)	0.055 (3)	−0.025 (3)	−0.016 (2)	0.003 (2)
C26	0.078 (3)	0.166 (6)	0.058 (3)	−0.049 (4)	−0.002 (2)	−0.006 (3)
C27	0.097 (4)	0.203 (7)	0.066 (3)	−0.092 (5)	0.000 (3)	0.006 (4)
C28	0.088 (3)	0.147 (5)	0.059 (3)	−0.075 (3)	−0.008 (2)	0.008 (3)

### Geometric parameters (Å, °)

**Table:**

I1—C25	2.091 (6)	C12—H12	0.9300
S1—C20	1.823 (4)	C13—C18	1.383 (5)
S1—C19	1.872 (3)	C13—C14	1.385 (5)
O1—C22	1.317 (5)	C13—C19	1.542 (4)
O1—C21	1.449 (5)	C14—C15	1.387 (5)
O2—C22	1.202 (5)	C14—H14	0.9300
C1—C2	1.384 (4)	C15—C16	1.363 (6)
C1—C6	1.392 (4)	C15—H15	0.9300
C1—C19	1.542 (4)	C16—C17	1.372 (6)
C2—C3	1.384 (5)	C16—H16	0.9300
C2—H2	0.9300	C17—C18	1.384 (5)
C3—C4	1.374 (6)	C17—H17	0.9300
C3—H3	0.9300	C18—H18	0.9300
C4—C5	1.371 (6)	C20—C21	1.498 (5)
C4—H4	0.9300	C20—H20A	0.9700
C5—C6	1.377 (5)	C20—H20B	0.9700
C5—H5	0.9300	C21—H21A	0.9700
C6—H6	0.9300	C21—H21B	0.9700
C7—C8	1.378 (4)	C22—C23	1.486 (6)
C7—C12	1.395 (4)	C23—C24	1.378 (7)
C7—C19	1.532 (4)	C23—C28	1.379 (6)
C8—C9	1.392 (5)	C24—C25	1.382 (6)
C8—H8	0.9300	C24—H24	0.9300
C9—C10	1.361 (5)	C25—C26	1.385 (8)
C9—H9	0.9300	C26—C27	1.367 (9)
C10—C11	1.375 (5)	C26—H26	0.9300
C10—H10	0.9300	C27—C28	1.384 (8)
C11—C12	1.379 (5)	C27—H27	0.9300
C11—H11	0.9300	C28—H28	0.9300
			
C20—S1—C19	105.86 (16)	C15—C16—H16	120.6
C22—O1—C21	117.5 (3)	C17—C16—H16	120.6
C2—C1—C6	118.2 (3)	C16—C17—C18	120.5 (4)
C2—C1—C19	121.6 (3)	C16—C17—H17	119.8
C6—C1—C19	120.0 (3)	C18—C17—H17	119.8
C3—C2—C1	120.7 (3)	C17—C18—C13	121.4 (3)
C3—C2—H2	119.6	C17—C18—H18	119.3
C1—C2—H2	119.6	C13—C18—H18	119.3
C4—C3—C2	120.2 (4)	C7—C19—C1	110.8 (2)
C4—C3—H3	119.9	C7—C19—C13	112.6 (2)
C2—C3—H3	119.9	C1—C19—C13	109.4 (2)
C5—C4—C3	119.7 (3)	C7—C19—S1	109.38 (19)
C5—C4—H4	120.2	C1—C19—S1	102.89 (19)
C3—C4—H4	120.2	C13—C19—S1	111.3 (2)
C4—C5—C6	120.5 (3)	C21—C20—S1	109.3 (3)
C4—C5—H5	119.8	C21—C20—H20A	109.8
C6—C5—H5	119.8	S1—C20—H20A	109.8
C5—C6—C1	120.7 (3)	C21—C20—H20B	109.8
C5—C6—H6	119.7	S1—C20—H20B	109.8
C1—C6—H6	119.7	H20A—C20—H20B	108.3
C8—C7—C12	117.8 (3)	O1—C21—C20	106.6 (3)
C8—C7—C19	122.0 (3)	O1—C21—H21A	110.4
C12—C7—C19	120.2 (3)	C20—C21—H21A	110.4
C7—C8—C9	120.8 (3)	O1—C21—H21B	110.4
C7—C8—H8	119.6	C20—C21—H21B	110.4
C9—C8—H8	119.6	H21A—C21—H21B	108.6
C10—C9—C8	120.6 (3)	O2—C22—O1	123.0 (4)
C10—C9—H9	119.7	O2—C22—C23	123.6 (4)
C8—C9—H9	119.7	O1—C22—C23	113.4 (4)
C9—C10—C11	119.5 (3)	C24—C23—C28	120.0 (4)
C9—C10—H10	120.3	C24—C23—C22	118.1 (4)
C11—C10—H10	120.3	C28—C23—C22	121.9 (5)
C12—C11—C10	120.4 (3)	C23—C24—C25	120.5 (5)
C12—C11—H11	119.8	C23—C24—H24	119.7
C10—C11—H11	119.8	C25—C24—H24	119.7
C11—C12—C7	120.9 (3)	C24—C25—C26	119.2 (5)
C11—C12—H12	119.6	C24—C25—I1	119.9 (4)
C7—C12—H12	119.6	C26—C25—I1	120.8 (4)
C18—C13—C14	117.4 (3)	C27—C26—C25	120.3 (5)
C18—C13—C19	120.9 (3)	C27—C26—H26	119.9
C14—C13—C19	121.6 (3)	C25—C26—H26	119.9
C13—C14—C15	120.7 (3)	C26—C27—C28	120.6 (5)
C13—C14—H14	119.7	C26—C27—H27	119.7
C15—C14—H14	119.7	C28—C27—H27	119.7
C16—C15—C14	121.2 (4)	C23—C28—C27	119.5 (6)
C16—C15—H15	119.4	C23—C28—H28	120.3
C14—C15—H15	119.4	C27—C28—H28	120.3
C15—C16—C17	118.8 (3)		
			
C6—C1—C2—C3	1.3 (5)	C2—C1—C19—C13	−104.0 (3)
C19—C1—C2—C3	176.8 (3)	C6—C1—C19—C13	71.4 (3)
C1—C2—C3—C4	−0.4 (5)	C2—C1—C19—S1	137.6 (2)
C2—C3—C4—C5	0.2 (6)	C6—C1—C19—S1	−46.9 (3)
C3—C4—C5—C6	−0.9 (6)	C18—C13—C19—C7	−98.5 (3)
C4—C5—C6—C1	1.9 (6)	C14—C13—C19—C7	79.5 (4)
C2—C1—C6—C5	−2.0 (5)	C18—C13—C19—C1	25.2 (4)
C19—C1—C6—C5	−177.6 (3)	C14—C13—C19—C1	−156.8 (3)
C12—C7—C8—C9	0.3 (5)	C18—C13—C19—S1	138.2 (3)
C19—C7—C8—C9	178.6 (3)	C14—C13—C19—S1	−43.8 (4)
C7—C8—C9—C10	−0.9 (6)	C20—S1—C19—C7	−42.7 (2)
C8—C9—C10—C11	1.2 (6)	C20—S1—C19—C1	−160.5 (2)
C9—C10—C11—C12	−0.8 (6)	C20—S1—C19—C13	82.4 (2)
C10—C11—C12—C7	0.1 (6)	C19—S1—C20—C21	133.8 (3)
C8—C7—C12—C11	0.1 (5)	C22—O1—C21—C20	−157.3 (4)
C19—C7—C12—C11	−178.2 (3)	S1—C20—C21—O1	59.9 (4)
C18—C13—C14—C15	−0.8 (5)	C21—O1—C22—O2	−3.5 (7)
C19—C13—C14—C15	−178.9 (3)	C21—O1—C22—C23	175.9 (3)
C13—C14—C15—C16	0.1 (6)	O2—C22—C23—C24	−1.8 (7)
C14—C15—C16—C17	0.8 (6)	O1—C22—C23—C24	178.8 (4)
C15—C16—C17—C18	−1.0 (6)	O2—C22—C23—C28	178.7 (5)
C16—C17—C18—C13	0.2 (6)	O1—C22—C23—C28	−0.8 (6)
C14—C13—C18—C17	0.7 (5)	C28—C23—C24—C25	−0.6 (7)
C19—C13—C18—C17	178.7 (3)	C22—C23—C24—C25	179.8 (4)
C8—C7—C19—C1	−113.4 (3)	C23—C24—C25—C26	−0.1 (7)
C12—C7—C19—C1	64.9 (3)	C23—C24—C25—I1	177.1 (3)
C8—C7—C19—C13	9.6 (4)	C24—C25—C26—C27	0.7 (8)
C12—C7—C19—C13	−172.1 (3)	I1—C25—C26—C27	−176.5 (5)
C8—C7—C19—S1	133.9 (3)	C25—C26—C27—C28	−0.5 (10)
C12—C7—C19—S1	−47.8 (3)	C24—C23—C28—C27	0.8 (8)
C2—C1—C19—C7	20.8 (4)	C22—C23—C28—C27	−179.7 (5)
C6—C1—C19—C7	−163.8 (3)	C26—C27—C28—C23	−0.2 (9)

## References

[bb1] Barbour, L. J. (2001). *J. Supramol. Chem.* **1**, 189–191.

[bb2] Bruker (2007). *APEX2* and *SAINT* Bruker AXS Inc., Madison, Wisconsin, USA.

[bb3] Sheldrick, G. M. (1996). *SADABS* University of Göttingen, Germany.

[bb4] Sheldrick, G. M. (2008). *Acta Cryst.* A**64**, 112–122. 10.1107/S0108767307043930 18156677

[bb5] Westrip, S. P. (2010). *J. Appl. Cryst.* **43**, 920–925.

[bb6] Zhu, X., Lu, P. & Ng, S. W. (2011). *Acta Cryst.* E**67**, o2475. 10.1107/S1600536811034180 PMC3200903 22059031

